# Bacterial Virus Forcing of Bacterial O-Antigen Shields: Lessons from Coliphages

**DOI:** 10.3390/ijms242417390

**Published:** 2023-12-12

**Authors:** Andrey V. Letarov

**Affiliations:** Winogradsky Institute of Micrbiology, Research Center Fundamentals of Biotechnology RAS, pr. 60-letiya Oktyabrya 7 bld. 2, Moscow 117312, Russia; letarov@gmail.com; Tel.: +7-903-137-01-52

**Keywords:** bacteriophage, host range, O antigen, *E. coli*, bacteriophage adsorption

## Abstract

In most Gram-negative bacteria, outer membrane (OM) lipopolysaccharide (LPS) molecules carry long polysaccharide chains known as the O antigens or O polysaccharides (OPS). The OPS structure varies highly from strain to strain, with more than 188 O serotypes described in *E. coli.* Although many bacteriophages recognize OPS as their primary receptors, these molecules can also screen OM proteins and other OM surface receptors from direct interaction with phage receptor-binding proteins (RBP). In this review, I analyze the body of evidence indicating that most of the *E. coli* OPS types robustly shield cells completely, preventing phage access to the OM surface. This shield not only blocks virulent phages but also restricts the acquisition of prophages. The available data suggest that OPS-mediated OM shielding is not merely one of many mechanisms of bacterial resistance to phages. Rather, it is an omnipresent factor significantly affecting the ecology, phage–host co-evolution and other related processes in *E. coli* and probably in many other species of Gram-negative bacteria. The phages, in turn, evolved multiple mechanisms to break through the OPS layer. These mechanisms rely on the phage RBPs recognizing the OPS or on using alternative receptors exposed above the OPS layer. The data allow one to forward the interpretation that, regardless of the type of receptors used, primary receptor recognition is always followed by the generation of a mechanical force driving the phage tail through the OPS layer. This force may be created by molecular motors of enzymatically active tail spikes or by virion structural re-arrangements at the moment of infection.

## 1. OPS Can Serve as Physical Barriers to Phage Adsorption

### 1.1. Exopolysaccharides and Surface Polysaccharides of Enterobacteria

**Figure 1:**
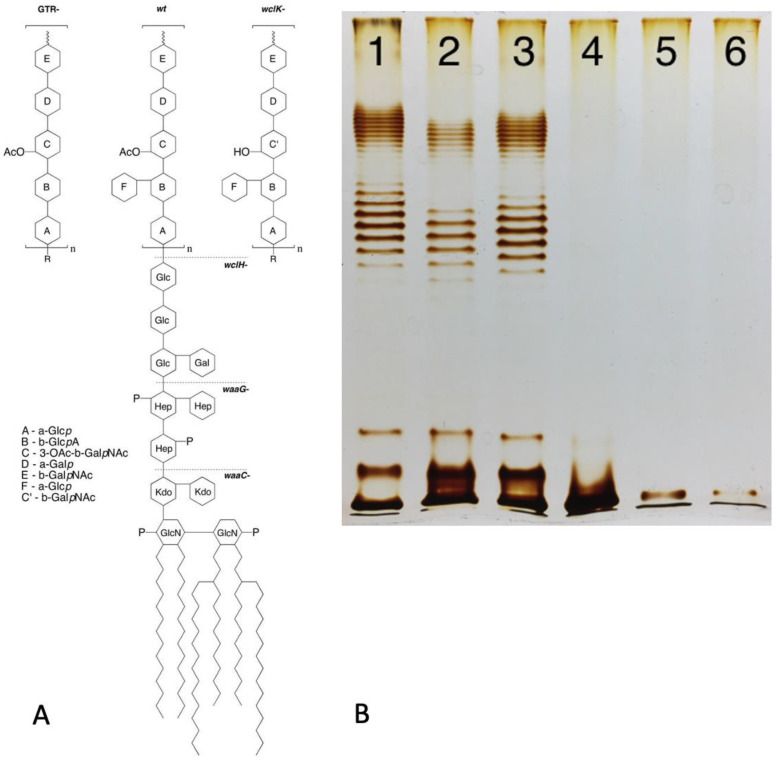
(**A**) Structure of LPS of *E. coli* 4s strain (O22 type with additional glucosylation) and its mutants lacking glucosylation (GTR-mutant) or O-unit O acetylation (*wclK-*). Dashed lines: the LPS structure in rough mutants (as exemplified by *wclH-*) and deep-rough mutants lacking outer (*waaG-*) or most of the inner core (*waaC-*). (**B**) LPS gel profile of the strains indicated in panel A: the wild-type strain (lane 1), non-acetylated OPS *wclK-* mutant (lane 2), non-glucosylated GTR- mutant (lane 3), rough *wclH-* (lane 4) and two deep-rough mutants waaG and waaC (lanes 5 and 6). Modified from [12], Creative Commons Attribution 4.0 International License (http://creativecommons.org/licenses/by/4.0/ accessed on 6 November 2023).

Bacterial cells often develop impressive surface-protecting structures such as capsules protecting individual cells, sheathes surrounding several cells or covering cell chains [1,2] and biofilms with large numbers of cells embedded in a common matrix [3]. These protective layers are generally described as exostructures, which can be mechanically removed without compromising cell viability or integrity. Most of these layers are of a polysaccharide nature and often referred to as capsular polysaccharides (CPS) or exopolysaccharides (EPS), although some capsule types instead consist of proteins [4] and the biofilm matrix often includes multiple types of polymers, including DNA or even filamentous phage particles (reviewed in [5]) in addition to EPS. The formation of such exostructures is optional and present only in certain species and strains of bacteria. The role of capsules and the biofilm matrix in the interactions of bacteria with their viruses, bacteriophages, have been extensively studied and recently reviewed elsewhere [6,7,8]. In Gram-negative bacteria, the actual outer surface of cells consists of an outer membrane (OM), which in most cases is covered by an additional polysaccharide structure termed an O antigen or O polysaccharide (OPS). An OPS is the outmost part of lipopolysaccharide (LPS) molecules [9] (Figure 1) that constitutes the bulk of the lipid material of the outer OM leaflet (while the inner OM leaflet consists mostly of phospholipids). This OPS (Figure 1) is attached to what is known as the core oligosaccharide (core-OS), which in turn is attached to lipid A, whose structure is highly conserved within bacterial species [9,10].

Structurally, OPS is a linear polysaccharide built of repetitive oligosaccharide motifs (O units). The O-unit backbone is generally one to six sugar residues in length. In some O serotypes, the O unit may contain lateral sugar residues or acetyl groups [9]. These lateral branches, however, apparently never extend beyond a single sugar residue. Both core-OS and OPS are synthesized by corresponding enzymatic pathways. The variability of core-OS synthesis genes is limited. For example, in *Escherichia coli*, only five core-OS types, referred to as K12, R1, R2, R3 and R4, have been described [11]. In contrast, OPS variability is much greater, with about 180 structural types (referred to as O serotypes) found in different *E. coli* strains [10].

### 1.2. OPS Synthesis

**Figure 2:**
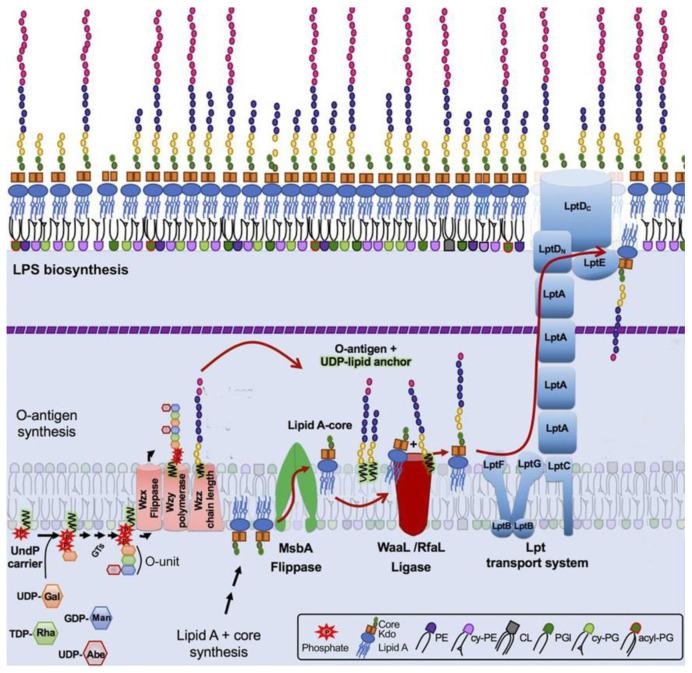
Model of lipopolysaccharide (LPS) synthesis with Wzy-dependent O-polysaccharide (OPS) polymerization and LPS transportation to the outer membrane in enterobacteria. LPS molecules are synthesized from two precursors, lipid A-core molecules and OPS, generated by two independent pathways. Then, LPS is assembled by the ligation of O antigens to lipid A-core molecules. OPS synthesis begins at the cytosolic side of the inner membrane (IM) by a sequential transfer of monosaccharide residues from sugar-nucleotide donor molecules to the undecaprenyl phosphate carrier (Und-PP) lipid to form a Und-PP-linked O unit. The Und-PP-O units are then flipped to the outer IM leaflet, polymerized by the Wzy OPS polymerase under the control of the Wzz protein regulating OPS chain length. Lipid A-core biosynthesis begins in the cytoplasm and continues in parallel with OPS synthesis at the IM inner leaflet. Next, the O-antigen and lipid A-core structures are joined with WaaL, also known as RfaL, to form one LPS superstructure. The Lpt complex spans the dual bilayers of the envelope and drives unidirectional LPS transport across the periplasm. Modified from [15], Creative Commons Attribution 4.0 International License (http://creativecommons.org/licenses/by/4.0/ accessed on 6 November 2023)).

The mechanisms of OPS biosynthesis have been extensively investigated and recently reviewed [9,10]. Most O antigens are synthesized via the Wzx/Wzy pathway (Figure 2). In this pathway, the O unit is assembled at the cytosol side of the cytoplasmic membrane (CM), being anchored in the CM by the adaptor lipid, Und-PP. The sugar residues comprising the O unit sequence are transferred to the adaptor sequentially by glucosyltransferases (GT) from the respective sugar nucleotides [13,14]. Once the O unit is completed, Wzx flippase translocates the Und-PP-O-unit precursor onto the periplasmic side of CM, where OPS is polymerized by Wzy polymerase. The Wzz protein controls the O-chain length. Finished OPS chains are transferred from Und-PP to vacant lipooligosaccharide (lipid A + core-OS) molecules by WaaL ligase. Complete LPS are then transported to the OM by the LOL transperiplasmic transportation system [13,14]. Interestingly, the number of O units is not tightly controlled, therefore yielding LPS molecules with different OPS lengths within a single cell. The distribution of LPS is uneven and features one to two maxims, often in the ranges of 12–17 and 25–35 O units. This bimodal LPS distribution can be visualized by SDS–polyacrylamide gel electrophoresis of LPS with silver staining [12] (Figure 1).

A much smaller number of OPS types are synthesized by the ABC transporter pathway. In this pathway, the linear OPS chain is assembled at the cytoplasmic side of CM and then Und-PP-OPS is transported to the periplasmic side of CM by the cognate ABC transporter [13,14].

### 1.3. Other Surface Polysaccharides of E. coli

Several less abundant surface polysaccharides are present in the order Enterobacteriales, which contains the genus *Escherichia*. The enterobacterial common antigen (ECA) is a linear polysaccharide built of repetitive units containing three residues: N-acetyl-d-glucosamine-(α1–}-N-acetyl-d-mannosaminuronic acid(β1–}-4-acetamido-4,6-dideoxy-d-galactose [16]. The dominant surface-exposed form of ECA is ECA_PG_ in which the ECA polysaccharide is linked by a phosphodiester bond to the unidentified lipid anchoring it in the outer OM leaflet. This ECA form may comprise up to 0.2% of dry cell weight. In addition to the ECA_PG_ form, ECA_LPS_ molecules may also be present in which the ECA polysaccharide is linked to the core-OS of some fraction of the LPS molecules instead of the OPS chains. The abundance of ECA_LPS_ is higher in rough strains, lacking OPS biosynthesis [16]. ECA biosynthesis is quite similar to Wzy/Wzx-dependent OPS synthesis with the repetitive units assembled at the inner CM side and the ECA chain polymerized at the periplasmic CM side by WzyE polymerase under the control of the chain-length regulation WzzE protein [17], with subsequent transfer of the ECA chain to the anchoring lipid and/or LPS molecules and translocation to the OM surface [16,18]. Besides ECA_PG_ and ECA_LPS_, the cyclic form of the ECA polysaccharides is present in the periplasmic space in at least some strains [16], but cyclic ECA is not surface-exposed and supposedly has no influence on host recognition by bacteriophages.

Recently, a new type of surface polysaccharide, NGR (from N4 glycan receptor), was discovered in *E. coli* [19,20]. This polysaccharide is secreted via the channel formed by the proteins NfrA and NfrB that were known earlier only as the phage N4 receptors [21]. Some exopolysaccharides such as the above-mentioned capsular polysaccharides, bacterial cellulose [22,23] and poly-β-1,6-N-acetylglucosamine [24] may retain a link to the OM surface at the moment of their secretion or thereafter, contributing to phage–host interactions (see below).

## 2. OPS-Mediated OM Protection

### OPS Shields the Outer Membrane Surface

The O antigen is highly immunogenic, and the extensive variability of OPS in different strains of *E. coli* and other pathogenic enterobacteria has been interpreted as a strategy to avoid bacterial clearance by the immune system [25]. Nonetheless, although OPS represent an Achilles heel when facing the adaptive immune response, they effectively protect the immediate OM surface from interactions with a variety of the molecules, thereby improving the fitness of bacteria under different conditions. The O antigen, for example, was demonstrated to block the binding of antibodies targeting the structures located underneath the OPS layer [26,27,28]. Interestingly, in *Salmonella*, the ability of OPS to block IgG binding to OM proteins depends on both protein size and the mean length of OPS chains [27]. Molecular modelling confirmed that the OPS chains in *Salmonella enterica* were too short to cover large trimers of OmpD proteins, whereas those same chains could cover the monomeric OmpA. This correlated well with the binding of the respective antibodies to the cell surface [27]. Note that, in the *S. enterica* O4 and O9 strains used in the aforementioned study, the OPS chains were about 6–11 O units long while the longer O antigens of many *E. coli* strains [12] may provide stronger protection even to large OM protein complexes. These data highlight the mechanical and non-specific protection provided by O antigens. This protection can be expected to be efficient against any bulky particles if no specific mechanisms to penetrate the shield are present. In good agreement with this conclusion, O antigens provide good protection against the bactericidal action of human or animal blood serum [29,30,31] and against the factors of innate immunity of insects [32] and plants [33]. The action of enzymes such as lysozymes can be also inhibited by O antigens [34,35]. O-antigen expression also reduces the phagocytosis of bacteria and their mortality due to monocytes and neutrophiles [36,37] or by amoeba [38]. The latter effects are most probably mediated by blocking the interaction of phagocytic cells with conserved molecular signatures of bacteria. This is because the O-antigen layer is relatively thin (~20 nm) and does not mechanically prevent the engulfment of bacterial cells. The protective features of O antigens make them important pathogenicity factors (reviewed in [10,17]).

## 3. Strategies of Host Cell Recognition by Bacteriophages and Properties of Phage-Resistant Mutants

Since the O antigen is able to prevent large molecules and particles from directly interacting with the OM, the structure should effectively protect the cell from bacteriophage infection. The fact that some bacteriophage and colicin receptors are efficiently blocked by O antigens of smooth strains of *E. coli* but exposed on their rough derivatives was demonstrated about 40 years ago [39]. A largely neglected fact, however, is that the widely used laboratory strains of *E. coli*, such as *E. coli* B and *E. coli* K12, are rough mutants lacking OPS production. In contrast, there is vast evidence in the literature reporting that bacteriophages use O antigen as receptors (reviewed in [40,41,42,43]).

Most tailed bacteriophages recognize at least two different receptors. The binding of the virus to the primary receptor does not trigger irreversible structural alterations to the virion. The recognition of the secondary receptor, in contrast, induces genome release. In some myoviruses, such as phage T4 (see below), the receptor is initially recognized by phage long tail fibers (LTF), which triggers baseplate rearrangement and tail contraction.

Different phages employ different host recognition strategies [40]: (I) Some phages recognize the secondary receptors directly (if the receptors are accessible); binding to primary receptors is optional for these viruses. This strategy (I) is used by many siphoviruses, such as phage T5, as well as by other phage morphological groups. If the primary receptor of a phage is the O antigen while the secondary receptor is an OM protein or LPS core-OS, this phage can effectively infect a large variety of rough host strains [12,44,45]. In contrast, smooth OPS-producing strains may be resistant or significantly less sensitive unless their OPS types are not recognized by phage RBPs. Accordingly, the phage host range may be significantly broader for rough derivatives compared to parental strains. Strategy II is employed mainly (but, again, not exclusively) by some podoviruses such as phage P22, in which recognition of the primary receptor is a prerequisite for interaction with the secondary receptor. The viruses employing strategy II and recognizing the O antigen as the primary receptor can only infect smooth strains. Rough derivatives, by contrast, are completely resistant to such phages. This holds true even if their cognate secondary receptors are intact and present at the OM surface. The strategy used by most (again, not all) myoviruses is closer to strategy I because the recognition of the first receptor triggers tail contraction. Even if an additional receptor is recognized after this event (such as the short tail fiber (STF) receptors in T4-like phages; see below), baseplate rearrangement and tail contraction are already irreversible, and if genome delivery fails, the virion is inactivated.

**Figure 3:**
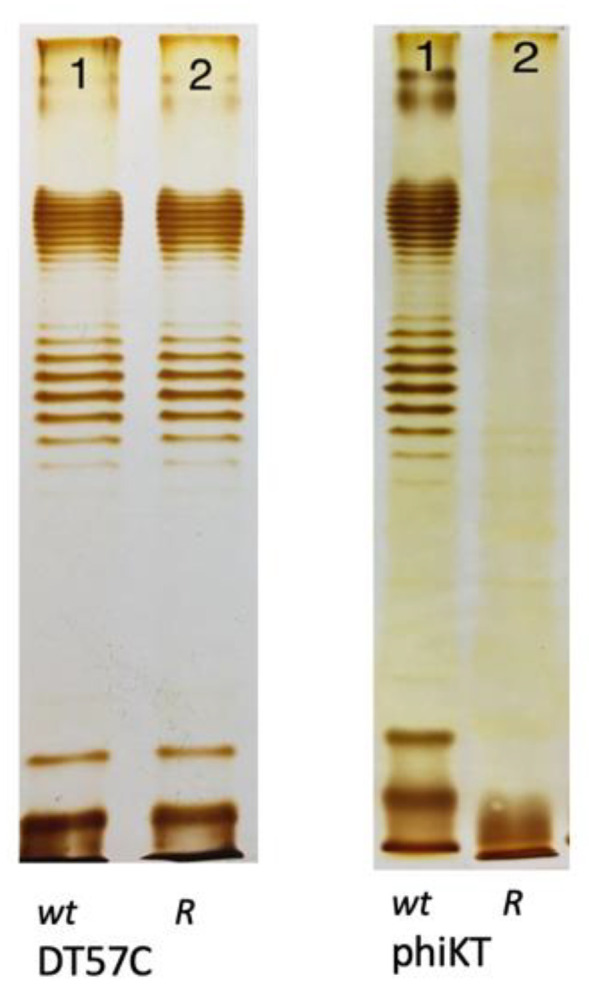
The LPS profiles of *E. coli* 4s mutants selected for resistance to a strategy I phage T5-like siphovirus DT57C and to a strategy II phage phiKT, a podovirus, distantly related to T7 (wt—wild type, R—resistant mutant). Images adapted from [12], Creative Commons Attribution 4.0 International License (http://creativecommons.org/licenses/by/4.0/ accessed on 6 November 2023).

Based on these differences, the O-antigen production status of host mutants selected for resistance to phages employing different strategies is expected to differ. The selective pressure from infection if a phage uses strategy I does probably not select for altered OPS phenotypes because removal of the primary receptor does not protect cells from phage attack. For example, the T5-like bacteriophages DT57C and DT571/2 [44] recognize OPS of several types using their lateral tail fibers (LTF). These phages have branched LTFs composed of two proteins, each harboring a receptor-binding domain. Thus, DT57C recognizes OPS of the types O22 and O81 of *E. coli* strains 4s and Hs3-104, whereas the DT571/2 phage binds O87 and O81 types of the strains HS1/2 and HS3-104 [44]; each phage may be able to recognize additional, as yet unidentified OPS types. Both phages recognize the identical secondary receptor BtuB [44] and can grow on the rough derivatives of the aforementioned strains [30,44] as well as on many other rough strains of *E. coli* [30,44,45]. Consecutively, the OPS production status of smooth host strain mutants selected by these phages under stringent conditions of plating onto phage agar is indistinguishable from the parental strains (Figure 3). All these mutants harbor mutations in the BtuB gene [44]. In theory, the secondary receptor may also be somehow functionally linked to the OPS production or surface expression. For example, if core-OS serves as the secondary receptor, then the phage can select for rough mutants lacking an outer core, and the OPS, even though the synthesis of the latter, may be not affected by the mutations (see Figure 1 right panel, lanes 5 and 6). Note, however, that such phage-host systems have not yet been described.

O-antigen-recognizing strategy II phages, in contrast, select for the rough phenotype. Even if the inactivation or modification of the secondary receptor is independent of the OPS (although the secondary receptors are seldom identified for such viruses), the synthesis of the latter requires specific metabolic pathways that represent larger targets for spontaneous mutations compared to, for example, an OM protein gene. Accordingly, the mutants are resistant to several strategy II types such as phiKT, Alt63 or G7C [12,46]. Here, again, there are exceptions to the general rule. For example, the G7C phage recognizes *E. coli* 4s OPS by means of enzymatically active tail spikes. As opposed to the vast majority of bacteriophage tail spikes exhibiting polysaccharide-depolymerizing activities (hydrolases or lyases) [41], the G7C tail spike is a deacetylase [47]. Therefore, one fraction of the host mutants selected for phage G7C resistance are true rough mutants with mutated genes of OPS backbone synthesis, whereas another fraction is represented by acetylation-deficient (*wclK-*) mutants [46]. The polymerization of the OPS from non-acetylated O-unit precursors is apparently less efficient, and the *wclK-* mutant showed a reduced surface expression of OPS [48]. These mutants remained sensitive to some of the O-antigen-dependent (strategy II) phages such as phiKT and Alt63. More recently, other phages harboring spikes with deacetylase activity towards OPS [49,50] and capsular polysaccharides of enterobacteria were discovered. These viruses may also select for two distinct phenotypes of resistant mutants. Finally, some phages may use OPS as one of several alternative primary receptors on a particular host strain [51], so they may also select for rough-resistant clones without being strategy II viruses.

The O-antigen expression status of enterobacteria is easy to determine using LPS profiling by polyacrylamide gel electrophoresis. Using the silver staining procedure optimized for preferential staining of polysaccharides yields high-quality LPS profiles (see example in Figure 1B) without labor-intensive LPS purification [12]. This enables resistant clone screening as a simple procedure to reveal the phage infection strategy, although, as exemplified above, potential alternative explanations of observed phenotypes should also be considered. The ability of strategy II phages to select for rough mutants probably contributes to the frequently reported decreased virulence of phage-resistant variants compared to parental genotypes [19,20]. Rough mutants also gain sensitivity to some strategy I phages that are not at all or poorly infective of parental strains (see below). Interestingly, in *Salmonella enterica*, the OPS length pattern controlled by phase-variable expression of the *opvAB locus* was shown to influence bacterial sensitivity to some OPS-specific bacteriophages. *opvAB*-ON phase cells featuring shortened OPS chains without a bi-modal distribution pattern were resistant despite the fact that OpvAB expression does not alter the O-unit structure [52,53]. Therefore, phages could select not for rough mutants but for mutants with different OPS length patterns. We also observed a similar effect with an RB49-like phage Whisky49 that selected *wzz*B-deficient mutants (our unpublished observations) on one of several *E. coli* strains resulting in an OPS pattern similar to that observed in *Salmonella* [53]. It is worth noting that Whisky49 phage is also able to recognize the OPS as a primary receptor on some of the host strains it infects ([51]; also see below)

## 4. How Strong Is the O-Antigen Barrier in Enterobacteria?

### 4.1. Blocking of Lytic Phage Infection by OPS

Until recently, the performance of bacteriophages potentially able to infect rough strains (such as domesticated *E. coli* K12) was seldom investigated on O-antigen-producing strains. Bacteriophage T5 was shown to recognize the polymannose O antigen of *E. coli* F by its LTFs [54,55] and to enhance the adsorption rate. Although it was possible to obtain O-antigen-dependent T5 mutants that could not infect the rough strains [56], the wild-type and even the *ltf^-^* mutants were able to form plaques on both rough hosts and the smooth *E. coli* F strain [54]. Therefore, the polymannose OPS did not effectively prevent the direct binding of the T5 straight tail fiber to the secondary receptor, FhuA. The same was reported for the *E. coli* F5 strain, producing the O28ab type of the O antigen [57], which could be infected by T5 and T5-like phages and by an LTF-depleted mutant of one of these viruses. Nonetheless, in contrast to the system phage T5—*E. coli* F, the efficiency of plating (EOP) of the phages, lacking RBPs specific to the OPS of the *E. coli* F5 strain, was decreased by five to six orders of magnitude. Interestingly, the mutant, lacking the LTF, performed slightly better compared to its parental phage. This most probably reflects steric constraints from bulky LTFs of the wild type, which were non-functional on this specific host [57]. At the same time, the host range of the several phages of DT57C species [58] strictly depended on an ability to recognize specific OPS types [44,45]. While no plaques were detected even with high phage dosage applied to the lawns of heterological strains, the EOP on the lawns of rough derivatives of these strains was comparable to that on the laboratory C600 or on the strains. This was specifically recognizable by the LTFs of the tested bacteriophage. The same pattern was also observed for another T5-like virus Gostya9 [57,59] and another siphovirus phage 9g as well as several other phages [30,57]. Interestingly, the *E. coli* 4s strain, which is sensitive to phage DT57C due to specific recognition of the O antigen by the phage LTFs, becomes totally resistant to this virus upon lysogenization by an O-seroconverting temperate phage Hf4s [60], which causes additional OPS glycosylation in the lysogens. Note, however, if rough variants of these lysogens are selected (for example, by a strategy II phage G7C), they return to sensitivity to phage DT57C despite the presence of the Hf4s prophage. The ability of OPS to restrict phage infection was recently confirmed by a systematic study by Maffei et al. [61]. The authors isolated a set of 68 *E. coli* K12 phages that well represented the known coliphage diversity; several standard model phages were also added to the study. Most of the phage types were completely or very significantly restricted on the *E. coli* K12 derivative, in which the production of the O16-type O antigen was restored by precise deletion of the IS element inserted into the *wbbL* gene in the conventional K12 strain (in total, 51 out of 74 tested phages were severely restricted by O16 OPS). However, some phages were able to grow effectively on the *wbbL+* strain. These included all 15 of the v5-like (Vequintarvirinae) isolates, phage N4, some T-even-related phages and certain others. All but 11 of the phages examined were also blocked on all three tested natural *E. coli* isolates producing the O antigen. It is noted that, in this assay, other factors, such as host antiviral immunity systems, may be partially responsible for the host range limitation. Nonetheless, it is noteworthy that 5 out of 16 Tevenvirinae isolates could infect at least one natural *E. coli* strain.

### 4.2. OPS Restricts Prophage Acquisition

The influence of the O antigen on prophage acquisition is poorly investigated. Plaque formation on bacterial lawns requires a relatively high infection efficacy. In contrast, a much smaller rate of effective phage adsorption may be sufficient to form detectable numbers of lysogens. Using the Stx-converting bacteriophage phi24B marked by an antibiotic resistance gene, James et al. [62] demonstrated that the lysogenization range by this virus, determined as a set of host strains in which antibiotic-resistant lysogens were formed upon phage contact with liquid cultures of bacteria, was much broader than the phage lytic activity spectrum. This phenomenon may be of primary significance because lysogenization by an Stx-converting phage makes the *E. coli* strain shigatoxigenic (STEC). This process is believed to be a major pathway of formation of new STEC lineages, many of which are very dangerous foodborne pathogens [63,64]. The O-antigen production status of lysogens formed under conditions similar to those described by James et al. ([62]) was recently investigated [30]. The authors determined that almost all of the lysogenic clones formed out of several environmental *E. coli* strains belonging to different O serotypes turned rough. At the same time, the presence of phi24B prophage did not abrogate the O-antigen production, at least in *E. coli* 4s. These findings suggest that the lysogens were mostly formed out of naturally occurring rough mutants present in the cultures exposed to phage phi24B. Interestingly, mutants selected by lysogenization by phi24B:cat having lost the OPS shield became sensitive to a number of virulent phages that were unable to infect the parental strains [30]. Accordingly, the formation of smooth O-antigen-producing STEC strains with phi24B-like prophages requires further investigation. Potentially, under certain conditions that differ from those of the above-mentioned experiments, phi24B-related viruses and other strategy I temperate phages non-equipped with specific molecular devices to penetrate the O antigen can nevertheless infect (at least at low probability) and lysogenize OPS-protected hosts. Among the conditions that may influence OPS-mediated restriction of lysogenization, the physiological state of the host cell appears the most probable. To my knowledge, the OPS protective efficiency has never been assessed on *E. coli* grown anaerobically and/or on starved cells that would better mimic the conditions in the human gut than actively growing aerobic cultures. Interestingly, the environmental *E. coli* isolates that were lysogenic by phages closely related to the phage lambda or by P2-like phages were not only immune to the phages encoded by their own prophages but also resistant to (unable to adsorb) these viruses [65]. The authors explain this finding by expected evolution of the resistance in lysogens that initially were only immune to phages. I, however, speculate that the tested strains could be completely protected by the O antigen or rendered almost non-penetrable for lambda-like or P2-like phages. Unfortunately, the mechanism of the resistance observed [65] was not analyzed in the study.

### 4.3. OPS-Mediated Protection in Other Species

The data cited above highlight the fact that the OPS-mediated protection of cells from bacteriophage attack is potentially a non-specific effect based on physical screening of receptors closer to the OM surface, such as OM proteins or LPS core-OS, from interaction with bacteriophage RBPs. Most of the environmental isolates of *E. coli* producing O antigens are, therefore, completely or almost completely (EOP < 10^−5^) protected from those phages lacking specific mechanisms to penetrate the OPS barrier. The efficacy of the O-antigen-mediated non-specific antiphage protection is poorly investigated in bacterial species other than *E. coli*, even within the order Enterobacteriales. Nonetheless, given such non-specific mechanisms of protection, significant levels of expression of sufficiently long OPS chains, especially with complex O-unit structure, should be equally effective in OM shielding with these other bacteria, as has been observed in *E. coli*. For example, in *Pseudomonas protegens*, the O antigen was found to protect the cell from insect immunity factors and can potentially be effective against the phages not able to specifically recognize the OPS [32].

## 5. Mechanisms Used by Bacteriophages to Penetrate the O-Antigen Barrier

The best-known mechanism of OPS penetration by bacteriophage is based on virion-associated, enzymatically active tail spikes that affect the OPS. The literature on phages recognizing O antigens and other surface polysaccharides of bacteria using enzymatically active tail spikes is extensive and has been recently reviewed elsewhere [66,67,68,69,70,71]. Most of these enzymes are polysaccharide depolymerases of hydrolase and lyase classes. Many phages, for example, the *S. enterica* temperate phage P22 [72] or related coliphage Hf4s [60], contain only one type of enzymatically active RBP (most often tail spikes), but some phages express multiple tail spikes that enable host infection with different types of the CPS, EPS or OPS. These RBPs frequently form branched structures including up to 14 different depolymerase RBPs, such as in *Klebsiella* giant myovirus φKp24 [73]. Although tail spike proteins with depolymerase activity have been found in all three major morphological types of tailed phages (myoviruses, podoviruses, siphoviruses), the way in which these proteins are involved in the multi-step cell recognition and infection process may differ in different virion organization variants.

### 5.1. Podoviruses: Cut or Pull?

The most popular model of enzyme-associated O-antigen and/or capsule penetration is based on the idea of partial removal of the barrier by enzyme(s), enabling RBP(s) to access the secondary receptor at the OM surface. “Drilling” a hole through the thick capsule of *E. coli* K1 cells by K1-specific podoviruses was first observed using electron microscopy by Lindberg as early as 1977 (cited in [74]). This concept is supported by the fact that purified recombinant, phage-derived tail spike proteins are able to remove the capsules or O antigens from the cell surface (see [70] for review). The enzymatic decapsulation of *Klebsiella*, *Acinetobacter* and some other bacteria by phage-derived proteins is demonstrably effective in treating experimental infections [69,70,71,75,76]. This is because removing the capsule makes the cells more vulnerable for the immune system of the macroorganism.

Nevertheless, certain facts challenge this simple barrier-breaking theory. Many bacteriophages bearing enzymatically active tail spikes are strategy II viruses, strictly dependent on the polysaccharide receptor for infection. Accordingly, the naked OM surface fully accessible for RBPs cannot be recognized by these viruses. The acapsular mutants of bacteria such as *Klebsiella* frequently acquire complete resistance to K-specific bacteriophages [75]. The nature of the conformational signal—that presumably must be generated upon interaction with the polysaccharide primary receptor to enable phage interaction with the OM surface (secondary receptor(s))—remains unclear in most cases. In the P22-like *Shigella* phage Sf6, the O antigen of the Y *Shigella* serotype [77] or, less efficiently, of the 2a_2_ serotype [78], serves as a primary receptor, whereby OmpA and OmpC are alternative secondary receptors [77]. At least in vitro, however, LPS alone or OmpA alone cannot trigger genome DNA release from the virions, but the two components added simultaneously cause efficient genome ejection [77]. Interestingly, the tail spikes of the phage Sf6 mutant selected for improved EOP on the 2a_2_ host featured a lower affinity to the 2a_2_ OPS [78]. So, although transient interaction with the O antigen is essential for subsequent recognition of the secondary receptor, a too strong binding to OPS may hinder subsequent infection events. The *Salmonella* phage P22 also uses the O antigen as a primary receptor. In this virus, purified LPS aggregates alone were sufficient to trigger genome release, but this was not the case with either soluble OPS or lipid A [79]. A cryo-EM study of phage P22 virion interaction with the cell surface revealed that the phage initially binds the cell obliquely through interaction of two neighboring tail spikes with the O antigen. This then brings the central tail needle into contact with the OM surface. The later interaction induces virion re-orientation into the orthogonal position with respect to the OM and subsequent DNA release [80]. The current model for P22 infection implies a role of mechanical force created by processive enzymatic cleavage of OPS by the tail spikes that push the central tail needle into the surface of the OM (or into micelles formed by isolated LPS in vitro). This force is sufficient to trigger virion rearrangements and subsequent DNA ejection [79,80].

The possible function of enzymatically active tail spikes as molecular motors may be further inferred from the data on host recognition by N4-like viruses. The coliphage G7C tail spike gp63.1 protein was shown to act as a deacetylase (esterase) instead of displaying the OPS depolymerization activity that is more common in bacteriophage RBPs. Though the OPS backbone remains intact upon gp63.1 treatment, the enzymatic activity is essential for infection of *E. coli* 4s [47]. The indirect data on the recombinant protein binding to the cell surface indicate that this deacetylation reaction is processive [47]. Bacterial rough mutants as well as mutants lacking O-unit O acetylation are completely resistant to phage G7C. Interestingly, in phage Alt63, which is almost identical to phage G7C, the esterase moiety of the gp63.1 tail spike is replaced by a classical depolymerase (lyase) domain [81]. Phage Alt63 remains a strategy II virus, but in contrast to phage G7C, phage Alt63 depends only on O-antigen expression, not also on OPS acetylation. Thus (remarkably), the infection mechanisms of N4-like phages G7C and Alt63 are fully compatible with both the OPS-degrading activity and deacetylase activity of their tail spikes without any noticeable modifications of other virion proteins.

Bacteriophage N4 has long been believed to be a strategy I virus, recognizing the NfrA protein receptor [82] using its so-called tail shaft protein, gp65 [83]. Recently, however, the previously unknown primary polysaccharide receptor, NGR (from N4 glycan receptor), was discovered [19,20]. The long-known NfrAB (from *N four receptor*) proteins were shown to be responsible for NGR secretion. Recognition and, probably, enzymatic degradation of this NGR polysaccharide are essential for N4 infection. Interestingly, the phage N4 was shown to infect multiple host strains, producing different OPS types [61]. Such a feature would be unexpected for a strategy I podovirus that depends on an OM protein for infection. The NGR receptor is present in small amounts on the cell surface because only about 45 copies of the NfrA protein (equal to five to six complexes) are present per cell [84]. At the same time, this polysaccharide is likely to be conserved among many *E. coli* strains, similar to enterobacterial common antigen (ECA). Nonetheless, interaction of one or few NGR chains should not automatically remove the screening of the OM surface by the O antigen. Thus, mechanical force generated due to NGR interaction (degradation or deacetylation) by some N4 RBP (most probably, gp66 tail spikes) may be responsible for driving the phage tail through the OPS layer.

**Figure 4:**
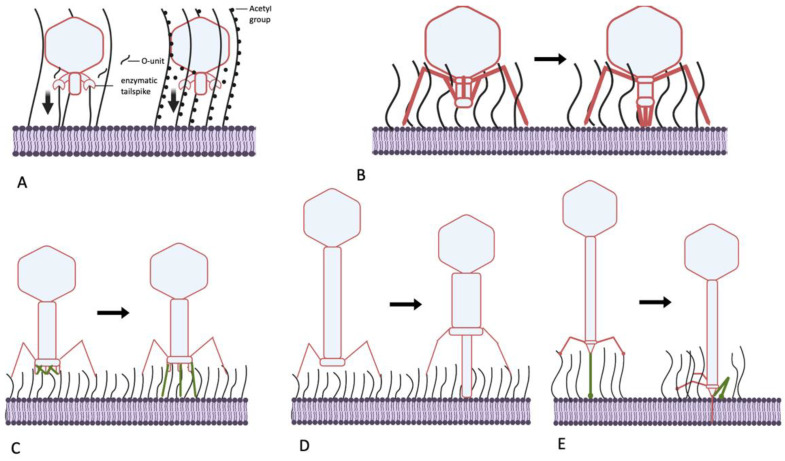
Mechanisms of generating mechanical force, helping bacteriophages to penetrate the O-antigen shield of *E. coli.* (**A**) Molecular motors, driven by processive polysaccharide depolymerization or deacetylation (for example, phage N4, P22-like viruses, G7C-like viruses). Vertical arrows: mechanical force generated; (**B**) movement of the tail components of certain podoviruses upon primary receptor recognition (as in phage SU10); (**C**) deployment of the STFs of T-even-related phages. (**D**) Piercing of the OPS layer by the tube of the contractile tail of long-tailed myoviruses (phage P1); (**E**) pulling the tail tube through the OPS by folding of the CTF in siphoviruses (for example, phage T5).

Summarizing the data presented above, I present the hypotheses that the podoviruses, by possessing the enzymatically active tail spikes, penetrate the O-antigen layer largely not by physically removing the OPS material from the virion’s way but instead by piercing this barrier using force that is generated by processive depolymerization or deacetylation of glycan receptors (Figure 4A). This could be OPS itself or other polysaccharides such as NGR or ECA, which, although less abundant on the cell surface, are better conserved. The latter can be. therefore. termed “key polysaccharides” because they help certain phages penetrate multiple different OPS types. Although this pulling mechanism seems to be more prevalent in podoviruses, other phage morphotypes, especially myoviruses (below), can adopt it or combine it with other mechanisms.

### 5.2. Can Podoviruses Push?

Interestingly, not all podoviruses that are able to infect O-antigen-producing strains possess enzymatically active tail spikes. For example, some T7-related (family Autographiviridae) bacteriophages instead recognize OPS as their primary receptor and behave as strategy II viruses. The phages phiKT and PGT2 [12,85], for example, recognize the O antigen using tail fibers carrying receptor-binding domains (RBD) that are similar to the RBDs of the lateral tail fibers of T5-related phage DT57C. These RBDs apparently lack any enzymatic activity. In phage T7, initial cell recognition by its tail fibers is followed by interaction of the tail nozzle protein with a so-far unidentified secondary receptor and subsequent exit of the internal capsid proteins (IPs) gp15, gp15 and gp16. These proteins extend to form a transperiplasmic conduit for the phage genomic DNA [86,87,88]. The proposed mechanism of this tail extension implies immersion of gp14 subunits into the OM lipid bilayer [88], which presumably requires tight contact of the distal tail tube (nozzle) protein with the OM surface. It is currently unclear how this mechanism can be adapted for “firing” from a ca. 20 nm distance if the internal protein release is activated when the nozzle remains at the surface of the OPS layer. Potentially, IP remodeling in phiKT-like phages may differ somewhat from that of its T7 analog, forming a slightly longer tail extension tube. The deployment of the tube creates a mechanical force sufficient for OPS penetration.

Another *E. coli* podovirus, SU10, a representative of the genus *Kuravirus*, also possesses tail fibers lacking any enzymatic activity. The distal domain of the SU10 long tail fibers is structurally similar to the phage T4 LTF needle, recognizing the primary receptor. The receptors of SU10 are not yet identified, but a cryo-EM study revealed in detail the interactions of this virus with the host cell surface [89]. The long tail fibers are not involved in any signal transduction, but the LTFs binding allows the central tail needle to interact with the OM surface, which probably triggers structural rearrangement of the tail. The tail needle apparently interacts with the host cell surface and separates from the nozzle protein, triggering the tail conformation change. Each of the six nozzle subunits contain four tandemly repeated so-called nozzle domains, forming a chain folded under the nozzle. These domain chains become extended towards the cell surface. The extended nozzle-domain chains interact with the short fibers, which rotate downwards. Eventually, the complex of six short tail fibers (STF) and six chains of the nozzle domains form an extended tail tube (see the supplementary video in [89]). The rearrangement process involving rigid STF rotation, nozzle domain extension and formation of additional interactions between these domains and STFs may also provide the mechanical power necessary to push OPS molecules away from the deploying tail (Figure 4B). Note that the overall tail extension is about 25 nm long [89], which corresponds to the expected OPS layer thickness. It is unclear if the STFs of bacteriophage SU10 recognize any specific secondary receptor or, instead, serve exclusively as structural elements for nozzle extension. After the extended nozzle contacts the OM, the internal proteins are ejected to form (presumably) a transperiplasmic channel that conducts the SU10 genome into the cell.

### 5.3. Myoviruses—Clutch and Push

The contractile tail machine of myoviruses created a paradigm of mechanical action generated by a virus particle. The general belief is that the main function of this tail contraction is to drive the internal tail tube through the cell wall and periplasm in order to provide a conduit to phage DNA transport [90]. The structure of some bacteriophage contractile tails, however, suggests that part of their forcing action is dedicated to breaking through the external structures, including through the O-antigen layer. Bacteriophage T4 and numerous related viruses recognize their primary receptors using their LTFs. In T4, the tip (needle domain) of the LTF forms the receptor recognition center [91], whereas in most other T-even-related phages, a monomeric RBP gp38 is present at the end of the LTF [92,93]. The binding of the several LTFs to the cognate receptor, which is initially a reversible binding, triggers the irreversible stage of the baseplate rearrangement from a hexagonal shape to a six-pointed star shape with simultaneous deployment of six STFs. These interact with secondary receptors to tightly fix the virion at the cell surface. The initial events of the baseplate rearrangement (specifically, the widening of the central gp6–gp7 ring) already trigger tail sheath contraction [90,94]. The tail sheath then acts as an extended spring that contracts, both driving the tail tube down and powering energic STF deployment. Phage T4 STFs are relatively long structures, and the baseplate of the infecting phage is held about 35 nm above the OM surface [95]. This distance appears to play no role in the infection of rough *E. coli* strains such as *E. coli* B—the typical laboratory host for phage T4 cultivation. This gap is, nonetheless, wide enough to avoid the need to bring the large and also flat baseplate through the OPS layer to the immediate surface of the OM.

Many T-even-related phages are able to infect *E. coli* strains producing O antigens of high protective ability [61,96]. Some RB49-like phages have recently been shown to recognize certain types of OPS as a primary receptor. These phages can even behave on some host strains such as *E. coli* F17 as strategy II viruses, although they are equally capable of infecting rough derivatives of other strains [51]. These findings indirectly indicate that RB49-like viruses activate their baseplate rearrangement before any RBP contact with the immediate OM surface. Interestingly, phage Brandy49, exhibiting the broadest host range within the investigated series of RB49-like viruses, uses some different, as yet unidentified mechanism that apparently allows its LTFs to penetrate different types of OPS to contact some receptor at the OM surface [51]. Regardless of the nature of the receptor recognized by T-even-related phages at the surface of the O-antigen-producing host, the mechanical force of their STF deployment appears to be the mechanism enabling their RBPs to penetrate the OPS barrier for tight phage attachment (Figure 4C). We currently lack a consistent explanation as to why the LTFs of phage Brandy49 can squeeze through the OPS whereas the LTFs of most other T-even-like phages cannot. Although the host specificity of T-even-like viruses is determined primarily by the monomeric RBP gp38 (except for T4 and some other viruses; see above), the internal regions of gp37, the trimeric protein of the distal LTF half, may also be involved in determining host range. Zhang and co-workers [97,98] reported that, in several closely related T-even-like phages, recombination with plasmids containing divergent fragments of gene 37 could swap the host specificity to that of the donor phage of the g37 sequence. Interestingly, the host ranges were tested using wild *E. coli* isolates, apparently producing protective O antigens. The involvement of gp37 in determining host range appears counter-intuitive because the genomes of the phages used in the work contained gp38 RBP homologs. Among possible explanations (beyond potential experimental errors), I speculate that gp37 may display several motifs capable of interacting with polysaccharides, though possibly with poor affinity and/or specificity. The sequential (starting from the baseplate-distal gp37 end) binding of such elements to OPS chains may be sufficient to submerge thin LTF into the O-antigen layer in a ratchet-like manner. This would bring the end of gp38 into contact with some receptor at the OM surface.

Another myovirus, phage P1, is known for its remarkably broad host spectrum [99,100]. Phage P1 exhibits a tropism-switching mechanism with two types of fiber genes, S and S’. At least with S’ fiber, the phage recognizes some of the O antigens in *E. coli* and *Shigella* as its only receptor [100], whereas S fiber binds the LPS core as its receptor [101]. Phage P1 has no STFs but its tail is much longer than phage T4’s (ca. 245 nm vs. 114 nm) [102]. Interaction of its LTFs with the receptor initiates tail contraction, and the tail sheath shrinks from 210 to 94 nm [102,103] (Figure 4 D). The phage is retained at the cell surface by extended LTFs, with a ca. 100 nm gap between the baseplate and OM [103]. This is sufficient for the tail tube to span the OPS layer using the force of the contracting tail (Figure 4D). The interaction of the S’ LTF with the OPS receptor is apparently strong enough to hold against the reaction force when the tail tube pierces the O antigen and other layers of the cell wall.

### 5.4. Siphoviruses—Grab and Drag

Although the molecular architecture of long non-contractile tails is well studied (reviewed in [104,105]), the function of a siphovirus virion during host cell attack is comparatively poorly understood. This also holds true relative to current knowledge on podovirus infection mechanisms. The only phage for which early infection events have been well described is the bacteriophage T5. Phage T5, along with most siphoviruses infecting enterobacteria, recognizes its secondary receptor using a central tail fiber (CTF). This fiber is equipped with a monomeric RBP, pb5 (in other siphoviruses, the RBD may be located directly on the trimeric CTF protein). In T5, the secondary receptor is the OM protein FhuA, although other T5-like viruses may infect through recognition of BtuB or FepA proteins [44,106]. T5 is also equipped with LTFs, but these fibers are attached rigidly [107,108], and the phage does not generate any conformation signal upon interaction with its OPS primary receptor (T5 is, therefore, a strategy I phage). As described above, many T5-like viruses rely on OPS recognition by the LTFs to infect O-antigen-producing host strains. At the same time, the link between LTF binding and subsequent penetration of the phage tail tip to the OM surface remains obscure.

The sequence of structural transformations of the T5 tail during infection of rough *E. coli* cells has been recently deciphered at atomic resolution [107,108]. The binding of the pb5 protein, located at the C end of the CTF protein pb4 trimer, to the FhuA receptor induces CTF conformation changes [109]. This causes the CTF to bend sharply such that its distal part (termed spike by Linares et al. [107]) interacts by its lateral loops with the fibronectin-like domains of the proximal part of pb4 and of the distal part of the baseplate hub protein pb3 (to which pb4 is attached) [107]. This interaction brings the distal and proximal ends of the CTF together (Figure 4E), tagging the tip complex to the OM surface directly next to the FhuA receptor molecule. After this event, the tape measure protein comes out of the tail to penetrate the OM bilayer and periplasm [107]. The CTF bending probably creates sufficient mechanical force to move the OPS molecules out of the way of the tail tip complex.

Summarizing all the data, I suggest the following model of infection of an O-antigen-protected host cell by a T5-like phage (such as *E. coli* 4s infection by the DT57C virus; [44]): The LTF binds to OPS reversibly, fixing the virion in an orientation that is suitable for infection. The CTF, being a narrow needle-like molecule, finds a gap in the O-antigen shield, benefitting from the Brownian motion of both the LPS molecules and the phage. After recognition of the OM protein receptor, the CTF bending generates a mechanical force sufficient to tug the tail tip through the O-antigen layer. Interestingly, in some T5-like phages infecting capsular strains of *Klebsiella,* the CTF protein is about twice as long as in T5, an adaptation compatible with the proposed model. Recently, the atomic-resolution structure of bacteriophage lambda’s tail was solved [110]. Although the alterations of this structure upon the cell surface binding were not experimentally analyzed, based on the structural features, the authors suggest that the mode of the lambda tail’s molecular machine action is similar to that of T5. Many other siphoviruses of enterobacteria have similar functional relationships between the CTF and LTFs (reviewed in [40,105]) but the mode of action of their CTF proteins could differ significantly given the lack of sequence similarity to the T5 pb4 or lambda J proteins.

## 6. Conclusions and Perspectives

The available data indicate that O antigens of most *E. coli* strains found in natural habitats make robust shields that protect the OM surface from direct interaction with large molecules or molecular complexes such as bacteriophages, antibodies, complement proteins or enzymes. In the case of phages, the protection afforded by many O-antigen types is sufficient to provide the cell with complete resistance to a virus, unless the latter is equipped with specific molecular mechanisms to penetrate this OPS barrier. Importantly, some phage strains, such as LTF-deficient mutants of T5-like phages, may serve as useful probe tools to test the protective function of the O antigen, in particular, bacterial strains, potentially under particular conditions. From an ecological perspective, the ability of the highly variable O antigen to determine and modulate the infectivity of bacteriophages may be a major factor influencing the diversity and dynamics of both phages and bacteria. For example, communities of commensal *E. coli* in the microbiomes of certain domestic horses include up to 1000 genetically distinct *E. coli* strains simultaneously present in a sample and having different profiles of sensitivity to the co-occurring coliphages [111,112]. In most of the phage–host systems isolated from this source, O antigens appear to play key roles in the phage sensitivity or resistance of bacteria (see [12] and refs therein, and refs 29, 58, 61 in this review). The role of the O antigen in controlling the spread of prophages has been recently demonstrated, but the data are too scarce to estimate the real-world significance of this phenomenon [30].

Bacteriophages employ a variety of mechanisms to penetrate the OPS shield and infect O-antigen-producing host strains. It should be highlighted that, despite the relatively small OPS layer thickness (about 20–30 nm), the data suggest that the protective effect of this structure has a non-specific nature and is due to mechanical shielding of the OM surface. Nevertheless, the recognition of OPS itself or any other receptor exposed outside of the OPS layer (for example, flagella, pili and conserved polysaccharides such as ECA, NGR or even bacterial cellulose [113,114]) does not automatically explain how a virus penetrates the OPS barrier. The analysis of the data on functional and structural detail of infection mechanisms of different coliphages has, however, enabled us to propose that the most if not all the mechanisms phages use to move through the OPS layer rely on the generation of a mechanical force (Figure 4). The specific mechanisms used to generate this force, however, may differ. They might range from molecular motors powered by processive depolymerization or deacetylation of polysaccharides by enzymatically active viral RBPs to clutching to certain available receptors and using the energy of the structural rearrangement of the phage particle to pass through the OPS layer.

Potentially, more variants of the force generation mechanism might still be discovered in coliphages and in viruses of other Gram-negative bacteria. At first glance, this model contradicts the widely accepted concept of the enzymatic breaking down of bacterial surface polysaccharide layers by virion-associated enzymes, although the significance of removing polysaccharide material from a patch of the cell surface may be more important in penetrating thick capsules of the extracellular matrix. In the case of O antigens, force generation appears to be a more common mechanism. This mechanism employs an intrinsic weakness of the OPS barrier, which is not only relatively thin but also composed of fluidly moving molecules. In an analogy to macroscopic objects, the O antigen is closer to the layer of hair of the skin than to clothes made of a tissue. Nevertheless, successful penetration of a phage through the OPS shield should never be considered to be a trivial event. The phenomenon of a wide-spectrum phage infecting multiple different host O serotypes should always be explained by identifying the mechanism enabling the respective virus to deal with a variety of structurally different but uniformly efficient barriers. Most probably, additional, elegant solutions developed during the evolution of bacterial predators will be discovered soon.

Finally, in some phage–host pairs, the OPS barrier provides bacteria with lower-protection yielding cells that are only partially resistant to virus attacks (see, for example, [55,57]). Accordingly, the trajectories of resistance development in a population of OPS-producing bacteria exposed to a phage may differ significantly from that observed in model systems with rough laboratory strains such as *E. coli* K-12 or B. The penetration of the OPS barrier may depend on the synthesis by the bacterial host of “key polysaccharides” such as ECA or NGR (see Section 3). This synthesis, in turn, may be modulated by cells’ physiological state and/or intercellular communications. Thus, the population-level adaptation strategies based on collective reactions of bacteria to phages (recently reviewed in [115,116]) may be also significantly impacted by the phage–OPS interplay in particular natural or experimental systems. Although data on the OPS-mediated OM screening in bacterial species other than *E. coli* are scarce, the non-specific nature of this phenomenon allows the tentative interpretation that such anti-phage protection may be common for many different bacteria with the Gram-negative cell wall type. This would make the phage–OPS interplay a major factor of bacteriophage ecology in many natural habitats.
